# A synchrotron X-ray spectroscopy study of titanium co-ordination in explosive melt glass derived from the trinity nuclear test†

**DOI:** 10.1039/c8ra10375e

**Published:** 2019-04-26

**Authors:** D. J. Bailey, M. C. Stennett, B. Ravel, D. E. Crean, N. C. Hyatt

**Affiliations:** Immobilisation Science Laboratory, Department of Materials Science and Engineering, University of Sheffield UK d.j.bailey@sheffield.ac.uk; National Institute of Standards and Technology 100 Bureau Drive Gaithersburg MD 20899 USA

## Abstract

The speciation of Ti in trinitite, the explosive melt glass derived from the Trinity Test of 16^th^ of July 1945, was investigated by X-ray absorption spectroscopy (XAS). Ti K-edge XANES showed that Ti was present in the Ti(iv) oxidation state for all samples. Fitting of pre-edge features by Gaussian functions and comparison with standards of known Ti coordination revealed significant variation in Ti coordination environment between samples. The variation of Ti coordination may be attributed to several factors including specific local chemistry and thermal histories of samples, in keeping with the highly heterogeneous microstructure of trinitite and the arkosic sand source material.

## Abstract 2

This work presents the first determination of Ti speciation and co-ordination in trinitite *via* the use of X-ray absorption spectroscopy.

## Graphical abstract

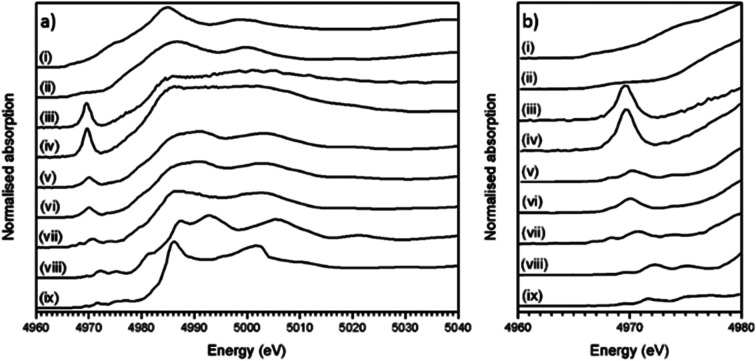

## 1 Introduction

Trinitite is an explosive melt glass derived from the Trinity Test that took place on the 16^th^ of July, 1945 at the White Sands Missile Range, New Mexico. The Trinity Test was the first detonation of a nuclear weapon and utilised a plutonium core and an implosion mechanism to achieve criticality.

The extreme heat generated by the resultant explosion (estimated average *T* = 8430 K, estimated extreme *T* = 3 × 10^7^ K (ref. 1 and 2)) melted or vapourised the vicinal Desert Sand and support structures. Rapid heating and cooling by afterwinds (estimated fireball duration ∼3.1 s^1^) resulted in the formation of a glassy product. The dominant morphology of trinitite specimens is 1–3 cm thick, green, glassy fragments with a smooth upper surface and a rough undulating lower surface.

Although predominantly green, high concentrations of elements, such as copper and iron, can change the colour of trinitite, resulting in ‘red’ and ‘black’ trinitite respectively. The majority of trinitite composition is drawn from minerals in the surrounding Desert Sand including: quartz (SiO_2_), microcline (KAlSi_3_O_3_), albite (NaAlSi_3_O_3_), muscovite (KAl_2_(AlSi_3_O_10_)(F,OH)_2_), actinolite (Ca_2_(Mg,Fe)_5_Si_8_O_22_(OH)_2_) and calcite (CaCO_3_).^3^ The inclusion of minority minerals such as titaniferous magnetite, rutile and barite has also been reported.^4,5^

The speciation and coordination of titanium in silicate melts has been found to be affected by the thermal history of the melt as well as other factors including composition and pressure during melting. Previous studies have used X-ray absorption spectroscopy (XAS) to determine the speciation of Fe in trinitite.^6^ This study presents the novel speciation of Ti in trinitite as determined by XAS.

## 2 Materials and methods

### 2.1 Trinitite samples and preparation

Trinitite specimens were supplied by the Mineral Research Company (one large 12 g piece and several small ∼1 g fragments) and the US Army (small ∼0.25 g fragments recovered from a sand grab sample obtained from the Trinity Test Site on the White Sands Missile Range).‡‡The Trinity Test Site was declared a national historic landmark in 1975 and it is illegal to remove material from this location. The sand grab sample used in this study was kindly provided by the Public Affairs Office of the White Sands Missile Range. The trinitite fragments had a variety of morphologies, but were consistent in appearance to previously studied examples.^1,3,4^ Also consistent with previously studied samples, numerous vesicles are observed from gas bubbles present in the melt.

Samples were prepared for bulk XAS by powdering aliquots from six small fragments. The powders were homogenously mixed with polyethylene glycol and pressed to form 13 mm diameter pellets. The Thin Section sample was prepared from the large trinitite specimen by sectioning and polishing to a ∼30 μm thickness. The specimen was sectioned vertically to preserve the smooth top and rough bottom of the sample in cross section, and was mounted in cold setting resin on a Spectrasil (Triad Scientific) fused quartz slide.

The average composition of the Desert Sand and trinitite was found by dissolving 0.5 g aliquots of representative material in hydrofluoric acid and then quantifying the constituents by inductively coupled plasma mass spectrometry (ICP-MS).

Elemental distribution within trinitite samples were studied by SEM-EDX using a Hitachi TM3030 electron microscope and equipped with a Bruker Quantax EDX detector. EDX data were analysed using Bruker Quantax software. Samples were prepared for SEM analysis by mounting in cold setting resin and polishing to an optical finish (1 μm) using SiC paper and progressively finer diamond pastes. Samples were sputter coated with carbon to reduce surface charging effects.

Gamma spectroscopy was used to characterise radionuclides contained within the remaining trinitite fragment samples using a Canberra BEGe detector. Spectra were gathered for 12 hours and the energy resolution of the detector was 0.15 keV.

### 2.2 XAS measurements

Samples were measured at the Ti K-edge using a conventional XAS setup at beamline X23A2, of the now decommissioned National Synchrotron Light Source, Brookhaven National Laboratory. Transmission mode measurements of the prepared samples were made alongside Ti standards (TiO, Ti_2_O_3_, TiO_2_ and CaTiO_3_). Incident (*I*_0_) and transmitted (*I*_t_) X-ray intensities were measured using ion chambers, energy calibration was performed with respect to XAS spectra measured with a reference ion chamber (*I*_r_) of a Ti foil placed after the transmission ion chamber in the beam path. Two different regions of the same Thin Section were measured in fluorescence mode. Fluorescence mode measurements were made using a four element Si drift detector. XAS spectra were measured from 30 eV below the Ti K-edge to 250 eV above, using a Si (311) monochromator. Data reduction and XANES analysis were performed using the program Athena.^7,8^

Fits to Ti pre-edge data of trinitite samples and standards were performed in the energy range 4965–4975 eV (0.3 eV energy resolution) to derive average oxidation state and co-ordination environment information.^9^ The XANES spectra were fitted following the method proposed by Waychunas, such fitting permitting direct comparison with other data gathered in previous studies regarding Ti co-ordination in minerals and glass melts.^9–14^ Data were normalised to a unit edge step using the Athena software package,^7^ and the rising edge background was fit using an arctangent function over the energy range 4960–5040 eV, as described previously.^15^ Gaussian components were then fit to the data to describe the components of the pre-edge features. The height and position of the weighted mean centroids of the functions were taken to be representative of the pre-edge feature.

## 3 Results and discussion

The composition of trinitite samples and Desert Sand, as found by ICP-MS, are shown in Table 1. As can be understood from Table 1, trinitite is primarily an alkali/alkaline earth aluminosilicate glass with considerable FeO and TiO_2_ additions. The ratio of non-bridging oxygens to tetrahedrally coordinated cations, NBO/T,^16^ is indicative of the connectivity of the glass network; a high NBO/T value would indicate a highly modified glass network with lower connectivity. Although there is not gross compositional variation between the two trinitite samples, a notable difference in NBO/T, calculated using eqn (1), was observed between the two trinitite samples.1
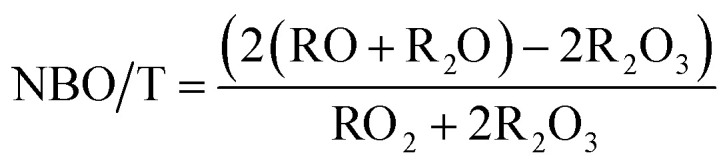


**Table 1:** Composition of trinitite and Desert Sand mol% by oxide^a^

Oxide	Desert Sand	Sample 1	Sample 2
SiO_2_	75.42	74.15	74.09
Al_2_O_3_	6.38	7.57	6.23
CaO	8.59	7.99	9.19
K_2_O	5.72	3.25	3.47
FeO	0.62	2.41	2.52
Na_2_O	2.69	2.18	2.07
MgO	0.34	1.81	1.75
TiO_2_	0.03	0.39	0.4
BaO	0.04	0.03	0.03
ZrO_2_	0.05	0.04	0.07
P_2_O_5_	0.04	0.07	0.06
MnO	0.01	0.05	0.06
SrO	0.02	0.02	0.02
PbO	0.01	0	0
CeO_2_	0.01	0	0
SO_4_	0.01	0.01	0.01
V_2_O_5_	0	0	0
Cr_2_O_3_	0	0.01	0.01
Other	0.02	0.02	0.02
Total	100	100	100
NBO/T	N/A	0.22	0.29

a Fe is known to be in the Fe^2+^ oxidation state (estimated error from measurement ± 2%). Results are restricted to major elements (0.01 mol%), full ICP-MS results are available in the ESI.

**Fig. 1:**
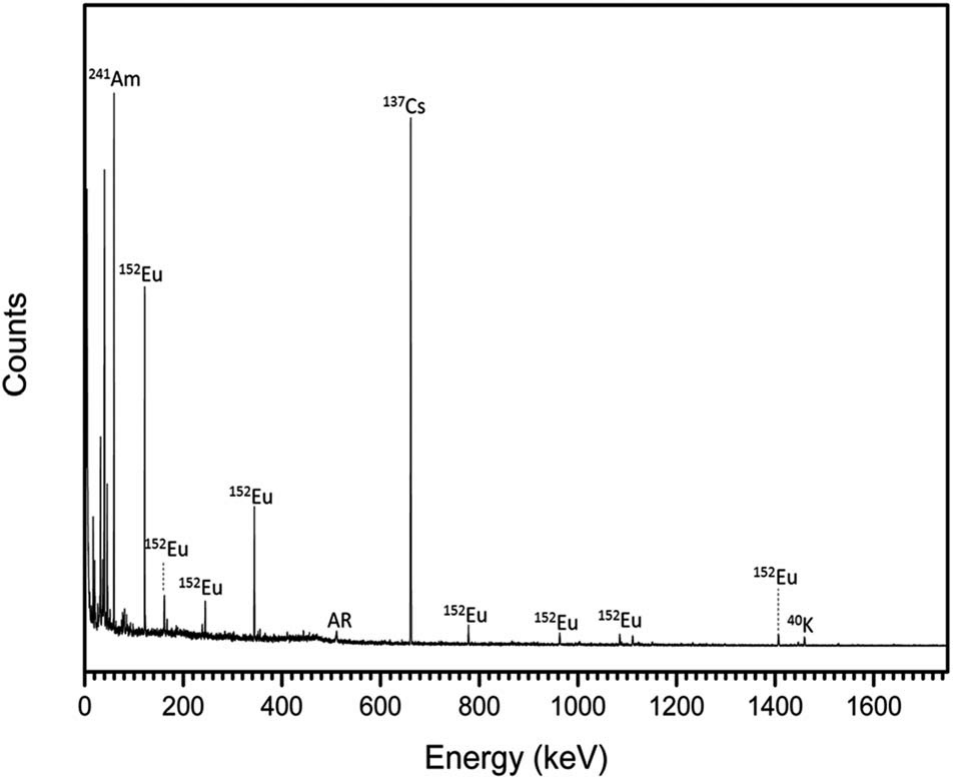
Gamma spectrum of representative trinitite fragments. AR = annihilation radiation caused by interaction of positrons and electrons (*E* = 511 keV).

Radionuclides contained within the trinitites were characterised by gamma spectroscopy. The resultant spectrum is shown in Fig. 1. As can be seen, the trinitite samples clearly contain ^241^Am, ^137^Cs and ^152^Eu indicating that the samples are the result of the detonation of a Pu-based fission device.^17^

**Fig. 2:**
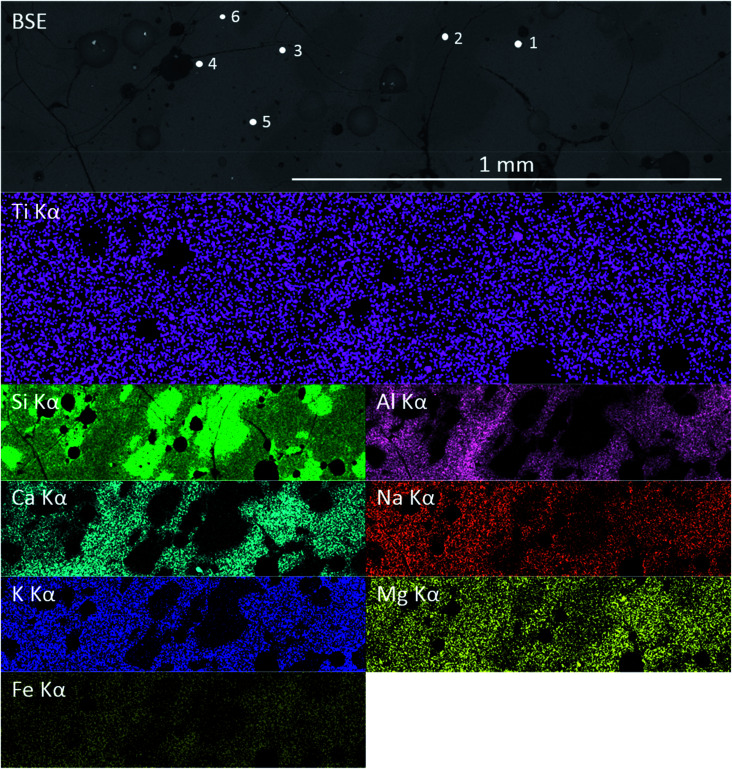
Elemental distribution within a representative sample of trinitite as determined by SEM-EDX.

**Fig. 3:**
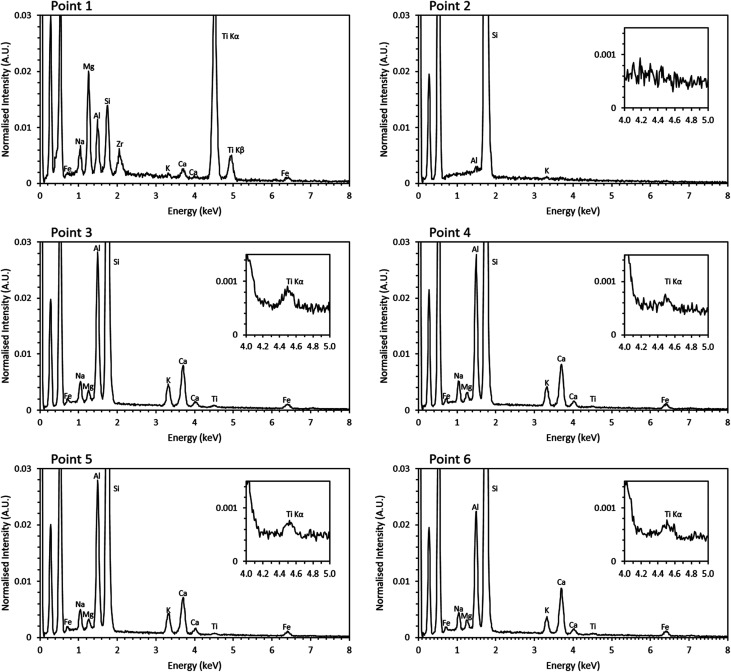
Point EDX spectra of locations denoted in Fig. 2. Inset, magnified Ti section of the spectra.

Previous studies have shown that trinitite is chemically inhomogeneous and actually comprises several glasses of varying composition and grains of unmelted, shock amorphised minerals.^5,18,19^ The results of SEM-EDX analysis of a representative sample are shown in Fig. 2 and 3 (Further EDX maps and spectra are available in the ESI†). As can be seen, elemental distribution varies across the sample with several distinct glasses: a high silica glass, an aluminium rich glass, a calcium rich glass and an alkali/alkaline earth aluminosilicate glass. Ti is present throughout the sample and, as evident in Fig. 2, local chemistry varies significantly. Spot EDX analysis (see Fig. 3) confirmed the variation in local chemistry as observed in Fig. 2. Point 1 is evidently a region highly enriched in Ti relative to the rest of the glass and point 2 is likely a grain of melted quartz. Points 3, 4, 5 and 6 are more similar in their composition however, it is apparent that there is local variation in chemistry; for example, point 6 is clearly enriched in Ca compared to points 3, 4 and 5. Local chemistry has previously been shown to affect the co-ordination of Ti in silicate melts and it is therefore possible to conclude that it is likely that Ti exists in several different co-ordination environments throughout the measured samples.^20^

**Fig. 4:**
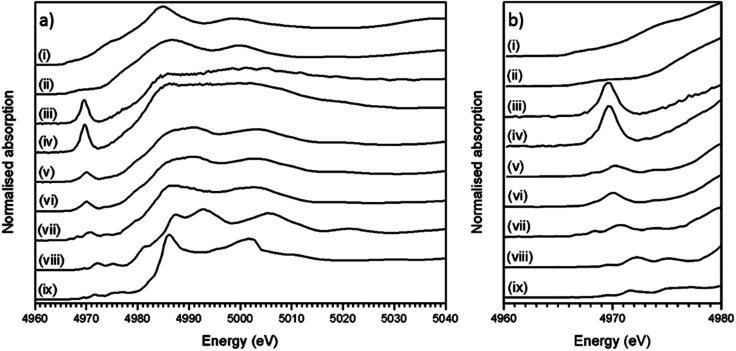
(a) Ti K-edge XANES spectra of trinitite samples, sand and standards; (b) detail of pre-edge XANES features. TiO(i), Ti_2_O_3_ (ii), Thin Section 1 (iii), Bulk Sample 1 (iv), Thin Section 2 (v), Bulk Sample 2 (vi), Desert Sand (vii), TiO_2_ (viii) and CaTiO_3_ (ix). Note the shift in pre-edge feature position for trinitite samples relative to the TiO_2_ and CaTiO_3_ standards. Thin section 1 and Thin Section 2 are different regions of the same Thin Section sample (Thin Section 1 is ∼14.7 mm closer to the top edge of the sample).

Fig. 4a shows the measured XANES spectra of the trinitite, Desert Sand samples and standards (TiO, Ti_2_O_3_ and CaTiO_3_). Fig. 4b shows a detailed view of the pre-edge region of the XANES spectra in Fig. 4a.

Ti K-edge XANES data show that the predominant oxidation state of Ti in all samples is Ti(iv), average for Ti(iv) standards *E*_0_ = 4984.1 ± 0.3 eV, Desert Sand *E*_0_ = 4984.3 ± 0.3 eV and average trinitite *E*_0_ = 4983.0 eV ± 0.3 eV (standard deviation = 0.4). This indicates that the oxidation state of Ti remains unchanged relative to that in the geological source material and is insensitive to the blast conditions. The trinitite samples, Desert Sand and the reference standards exhibit a distinct pre-edge feature at ∼18 ± 3 eV before the edge step.^11^ The pre-edge feature is attributed to transitions from Ti 1 s to bound Ti 3d/O 2p final states that reflect the hybrid Ti 3d–O 2p hybrid states in titanium compounds. A 1s to 3d transition is forbidden due to dipole selection rules (Δ*l* = ±1) however, this rule is relaxed when Ti is located in a non-centrosymmetric co-ordination environment and p–d mixing occurs.^9^ Waychunas and Farges have both demonstrated that the pre-edge position and intensity are a direct function of p–d mixing and consequently indicative of the Ti co-ordination environment.^9,11^ As can be seen from Fig. 4b, there is significant variation in the position and height of the pre-edge features indicating the existence of differing Ti co-ordination environments. Damping of the post-edge XANES features in the trinitite samples studied (see Fig. 4a) is a result of random phase decoherence and multiple scattering paths and is symptomatic of atomic disorder, typical of glasses.

**Fig. 5:**
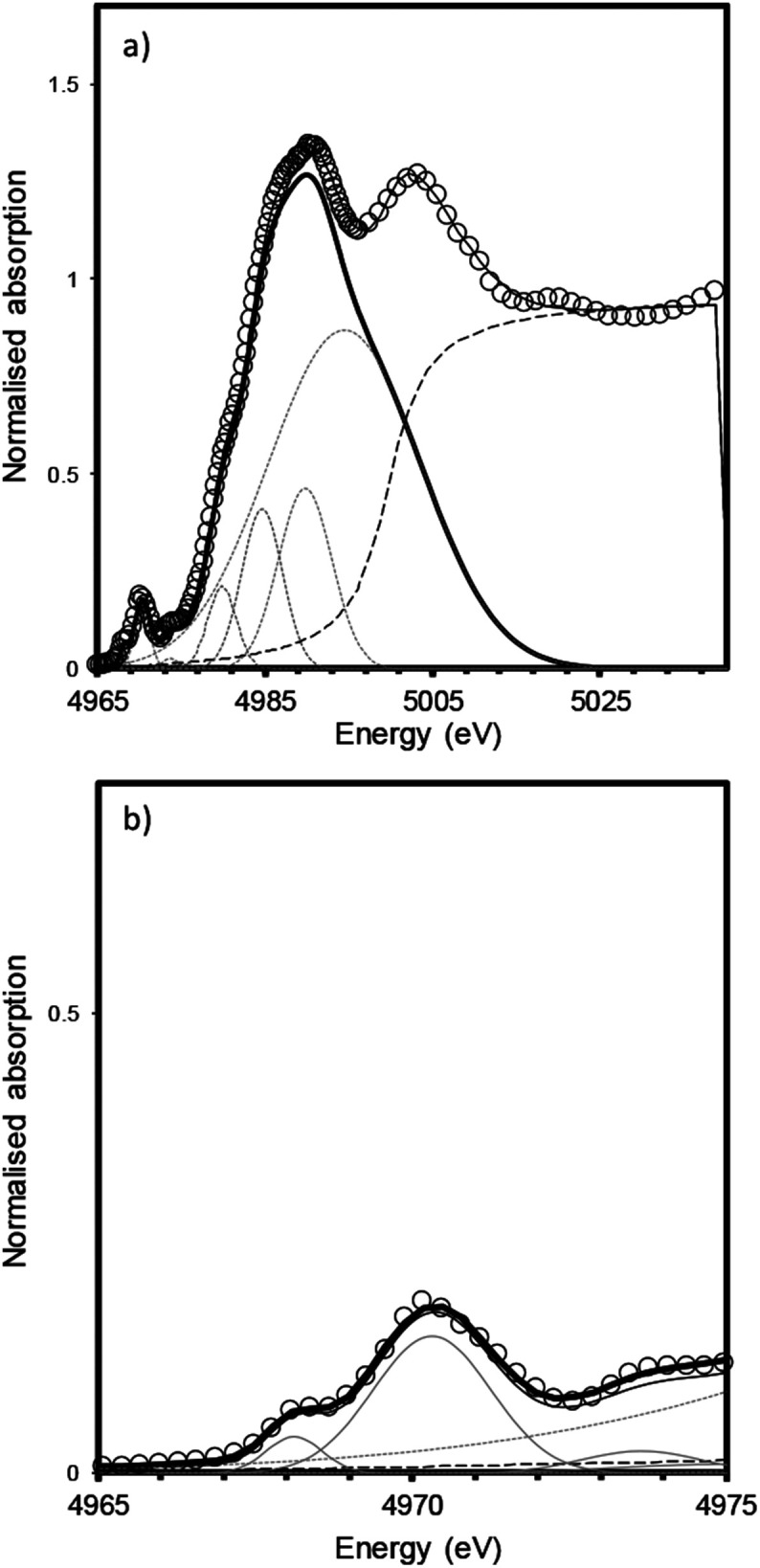
Fitting of XANES features for a bulk trinitite sample (Bulk Sample 2) measured in fluorescence mode. (a) XANES region (b) pre-edge features. The data were fit using a combination of Gaussian and arctangent functions (grey lines); data are shown by open symbols, fit is shown by a solid black line; methodology follows that of Waychunas.^9^

The measured XANES spectra were fitted according to the method used by Waychunas.^9^ Gaussian functions were fitted to the pre-edge envelope and the height and position of the weighted mean centroids of these functions were taken to represent the overall height and energy of the pre-edge feature. Fitted values are given in Table 2, an example fit is shown in Fig. 5. Although there have been recent advances in the modelling of XAS spectra by density functional theory (DFT), the large number of different elements and the mixture of numerous amorphous phases that constitute trinitite precluded the use of DFT to elucidate further details with regards to the local structure of Ti. Similarly, Principal Component Analysis (PCA) has become a method frequently used to study XAS spectra however, satisfactory analysis and assignment of spectral components to specific coordination environments requires a full suite of standards with which to compare samples of unknown coordination environment. Using the method of Farges *et al.* allowed experimental data to be compared with previously characterised and published standard spectra.

**Table 2:** Normalised pre-edge centroid height and energies of fitted Ti XANES spectra

Sample	Energy (eV)	Normalised pre-edge height
Thin Section 1	4969.6	0.56
Bulk Sample 1	4969.6	0.58
Thin Section 2	4970.2	0.21
Bulk Sample 2	4970.5	0.17
Desert Sand	4971.1	0.18
TiO_2_	4971.5	0.11
CaTiO_3_	4971.6	0.11
Precision	±0.3	±0.03

**Fig. 6:**
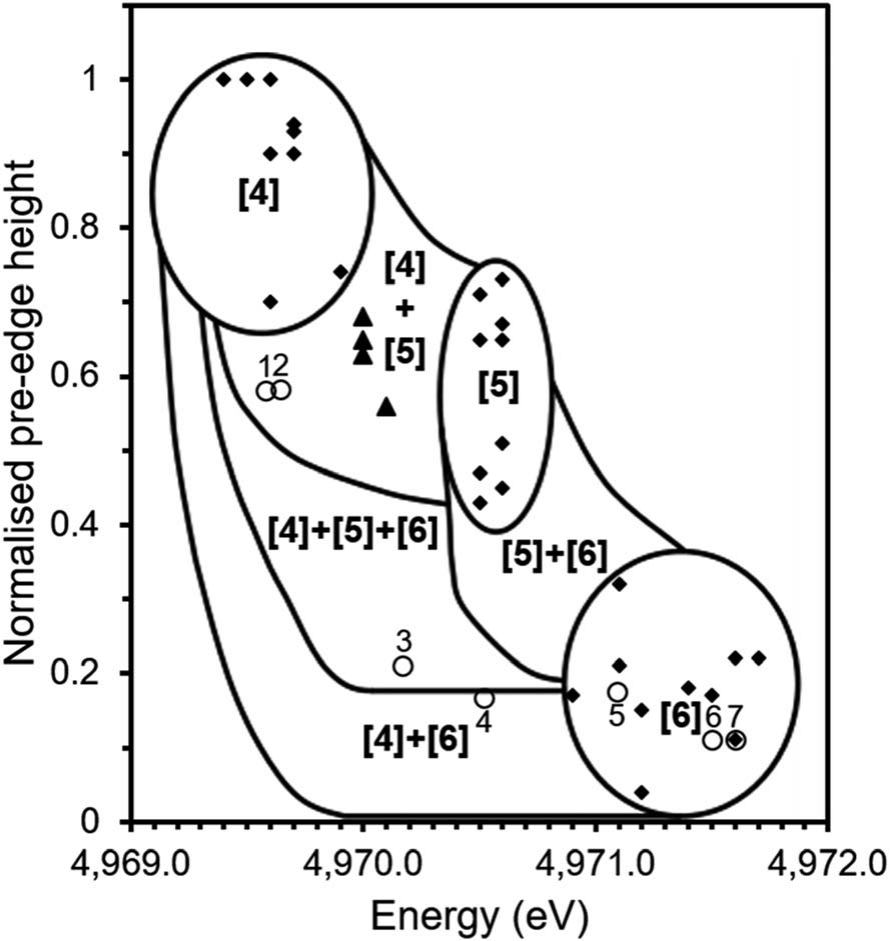
Ti co-ordination environment as speciated by pre-edge energy position and normalised height of pre-edge feature in Ti K edge XANES. Solid squares show data for mineral standards from previous studies;^11^ solid triangles show data for tektites from previous studies^14^ and open circles show data from this study for: Thin Section 1 (1), Bulk Sample 1 (2), Thin Section 2 (3), Bulk Sample 2 (4), Desert Sand (5), TiO_2_ (6) and CaTiO_3_ (7). Adapted from Farges (1997).^14^

Fig. 6 shows the correlation of pre-edge height and energy with co-ordination environment of Ti bearing standards (solid diamonds) and that of tektites (solid triangles) as reported by Farges *et al.*^10,14^ together with data from the current study (open circles). Distinct zones exist for 4, 5 and 6-fold Ti co-ordination with adjacent regions assigned from analysis of mechanical mixture of titanium compounds with Ti in different site geometries *i.e.* a mixture of 4 and 6-fold co-ordination.^10^

The determined pre-edge height (0.11 a.u.) and energy of (4971.6 eV) of the pre-edge feature in CaTiO_3_ is identical to that reported in previous investigations (4971.6 eV, 0.11; point 7, Fig. 6), it was therefore concluded that the measured data were consistent with the literature and it was possible to compare our data with that of previously reported standards measured using comparable X-ray optics.^11^ As can be seen in Fig. 6, the trinitite samples present a range of average Ti co-ordination environments.

Thin Section 1 and Bulk Sample 1 (data points 1 and 2 respectively) lie within the region associated with mixed four and five-fold co-ordinate Ti. Thin Section 2 (data point 3) lies in the region associated with mixed four, five and six-fold co-ordination, Bulk Sample 2 (data point 4) contains a mixture of four and six-fold Ti. The sand sample (data point 5) is located within the six-fold co-ordinated region indicating that the average co-ordination of Ti before the blast was six-fold, consistent with the reported presence of rutile.^5,6^

The region occupied by Thin Section 1 and Bulk Sample 1 is also that occupied by the tektites studied by Farges *et al.*^14^ Tektites are natural glasses formed by the impact of extra-terrestrial bodies and the melting of vicinal materials and it has previously been observed that trinitite is similar in morphology to some tektites.^21^ A study of natural glasses and tektites by Farges *et al.* (1997) found that the dominant co-ordination environment of Ti is five-fold in silicate melts, however, there is a significant amount of four-fold Ti in the most polymerised systems such as rhyolitic glasses and tektites. The co-ordination environment of Ti has been found to be strongly dependent on the ratio of Ti to non-bonding oxygens with glasses of lower ratios, such as tektites (NBO/T = 0.08–0.13), yielding more four and five-fold Ti.^14^ This would suggest that the trinitite glass of Thin Section 1 and Bulk Sample 1 is more highly polymerised than the trinitite of Thin Section 2 and Bulk Sample 2. This possibility is supported by the observed variation of NBO/T between the trinitite samples studied by ICP-MS, as shown in Table 1.

Ti coordination in synthetic, natural and impact glasses is known to be influenced by glass composition, pressure and the rate at which the glass is cooled.^13,20,22^ The chemical inhomogeneity of trinitite may explain the observed variation in Ti coordination. Trinitite composition is drawn from a range of minerals including alkali and alkaline earth bearing feldspars and, due to insufficient equilibration time, there is significant variation in the concentrations of these elements within specimens.^5,18^ Dingwell *et al.* (1994) found that Ti in alkaline earth bearing silicates had a higher average coordination number than Ti in alkali silicates.^20^ SEM-EDX analysis (see Fig. 2) has shown that local chemistry varies significantly across trinitite samples and, consequently, it is possible that Thin Section 2 and Bulk Sample 2 may contain a higher relative concentration of alkaline earth cations and hence have Ti in a higher average coordination number. High pressure has also been found to increase the coordination of Ti in melts,^22^ possibly indicating variation in pressure conditions during trinitite formation. Additionally, Ti coordination may be affected by the cooling rate from the melt temperature to ambient temperature.^13^ Comparisons of quenched and unquenched melts found that observed pre-edge height was greater in quenched samples, indicating retention of a greater degree of four-fold Ti. The higher normalised height and lower energy position of the pre-edge features of Bulk Sample 1 relative to Bulk Sample 2 may show that it was cooled more rapidly and retained a greater proportion of four-fold Ti. Another possibility that may explain the variation observed between the Bulk Samples is that Bulk Sample 2 was under greater pressure than Bulk Sample 1 at the time of formation and as result formed a greater degree of six-fold coordinated Ti. Cooling rate considerations were found to not apply to the two Thin Section measurements. Fig. 6 shows the pre-edge feature associated with the location of Thin Section 1 (within the Thin Section specimen interior) is consistent with a lower average Ti co-ordination number compared to the pre edge feature corresponding to location of Thin Section 2 (near the upper surface of the Thin Section specimen interior). If this trinitite were formed in a single major deposition event, the exterior would be expected to have cooled more rapidly due to afterwinds that followed the blast, resulting in a lower average co-ordination number for Ti at the surface, compared to the interior. Depletion of alkalis from the surface, by volatilisation, would result in a lower NBO/T ratio, and a lower average Ti co-ordination number relative to the interior. This is contrary to our observation, but could be rationalised by the local variation in chemical composition being of crucial importance, or a hybrid formation mechanism as proposed by Weisz *et al.* (2017).^23^ Further multi-element micro-focus XAS and XRF studies of this material would assist in understanding the spatial variation in Ti speciation as a function of the local chemical composition.

## 4 Conclusions

The speciation of Ti in trinitite was investigated by X-ray absorption spectroscopy. Ti was consistently present in the Ti(iv) oxidation state however, the coordination environment was found to be inhomogeneous. The variation in Ti coordination could be as a result of numerous factors with variations in local chemistry, network polymerisation and thermal history all possible contributors. The variation observed in this study is reflective of the diverse source material and extreme formation conditions of trinitite and serves to highlight the highly heterogeneous nature of melt material produced by nuclear weapons tests. Nevertheless, further micro-focus XAS and XRF studies of trinitites, linking element speciation to spatial disposition and local chemistry, may shed further light on the formation mechanism of this fascinating material.

## Conflicts of interest

There are no conflicts of interest to declare.

## Supplementary Material

RA-009-C8RA10375E-s001
