# Supplementary material for: A synchrotron X-ray spectroscopy study of titanium co-ordination in explosive melt glass derived from the trinity nuclear test
Source: RSC Adv. 2019 Apr 26;9(23):12921–7. doi: 10.1039/c8ra10375e (PMC9063809; doi:10.1039/c8ra10375e)
Supplement: RA-009-C8RA10375E-s001 [file RA-009-C8RA10375E-s001.pdf]

Electronic Supplementary Material (ESI) for RSC Advances.
This journal is © The Royal Society of Chemistry 2019

A Synchrotron X-ray Spectroscopy Study of Titanium Co-ordination in Explosive Melt Glass Derived From the Trinity Nuclear Test: Supplementary Materials

ST1: Raw ICP-MS data for desert sand and trinitite samples (mg.kg^{-1}).

	Desert Sand	Trinitite 1	Trinitite 2
Si	240000	260000	260000
Al	39000	51000	42000
Ca	39000	40000	46000
K	39000	24400	26100
Fe	3900	16800	17600
Na	14000	12500	11900
Mg	950	5500	5300
Ti	181	2310	2370
Ba	605	535	540
Zr	530	480	825
P	253	514	442
Mn	87.9	356	398
Sr	180	215	235
Pb	135	15	45
Ce	205	86	65
S	53	38	35
V	6.4	43	41
Cr	5.2	35	36
Cu	4.1	29	24
Li	7.4	15.2	16.5
Zn	14.4	19.5	16.1
Ni	3	18.9	18.8
La	16.8	26.1	25.7
Y	2.3	13.9	5.9
Nb	5.2	24.4	15.2
Co	<2	6.1	6.2
Be	5.6	1.4	1.5
Hf	16.3	14.9	26.2
U	1.65	2.53	2.98

ST2: Composition of desert sand and trinitite samples (mol% by oxide, 5 d.p.)

	Desert Sand	Trinitite 1	Trinitite 2
SiO_2	75.42311	74.14623	74.09303
Al_2O_3	6.37887	7.56958	6.2293
CaO	8.58898	7.99392	9.18641
K_2O	5.71847	3.24659	3.4703
FeO	0.6164	2.40952	2.52245
Na_2O	2.68746	2.17744	2.07144
MgO	0.34499	1.81248	1.74532
TiO_2	0.03338	0.38653	0.39628
BaO	0.03889	0.0312	0.03147
ZrO_2	0.05128	0.04214	0.07238
P_2O_5	0.03605	0.06646	0.05711
MnO	0.01412	0.0519	0.05798
SrO	0.01813	0.01965	0.02147
PbO	0.00575	0.00058	0.00174
CeO_2	0.01291	0.00492	0.00371
SO_4	0.01459	0.00949	0.00874
V_2O_5	0.00055	0.00338	0.00322
Cr_2O_3	0.00088	0.00539	0.00554
CuO	0.00057	0.00366	0.00302
Li_2O	0.00471	0.00877	0.00951
ZnO	0.00194	0.00239	0.00197
NiO	0.00045	0.00258	0.00256
La_2O_3	0.00053	0.00075	0.00074
Y_2O_3	0.00011	0.00063	0.00027
Nb_2O_5	0.00025	0.00105	0.00065
CoO	0.0003	0.00083	0.00084
BeO	0.00548	0.00124	0.00133
HfO_2	0.00081	0.00067	0.00117
U_3O_8	0.00002	0.00003	0.00003
Total	99.99998	100.00000	99.99998

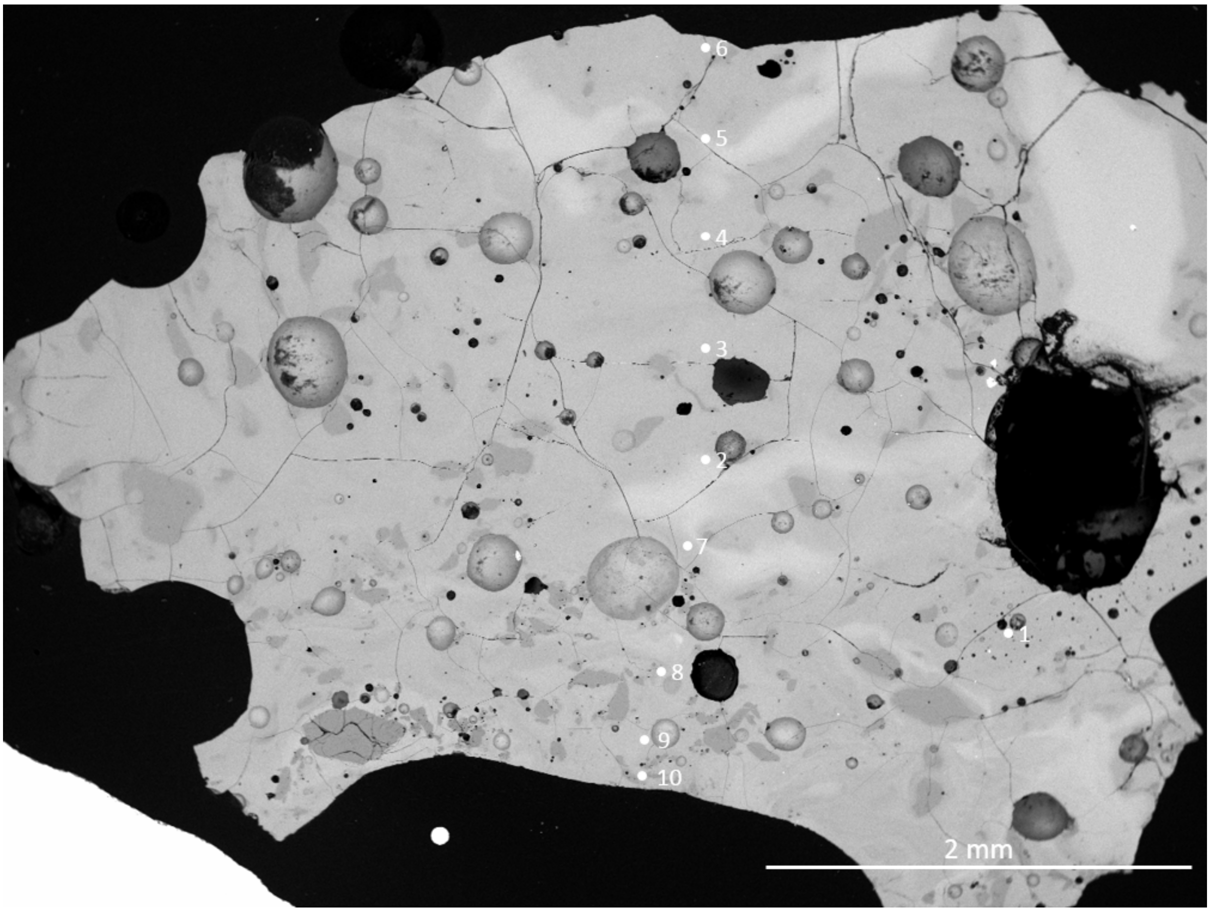

SF1: Backscattered electron micrograph of a trinitite specimen. Numbered points indicate the location of individual spectra shown in SF2.

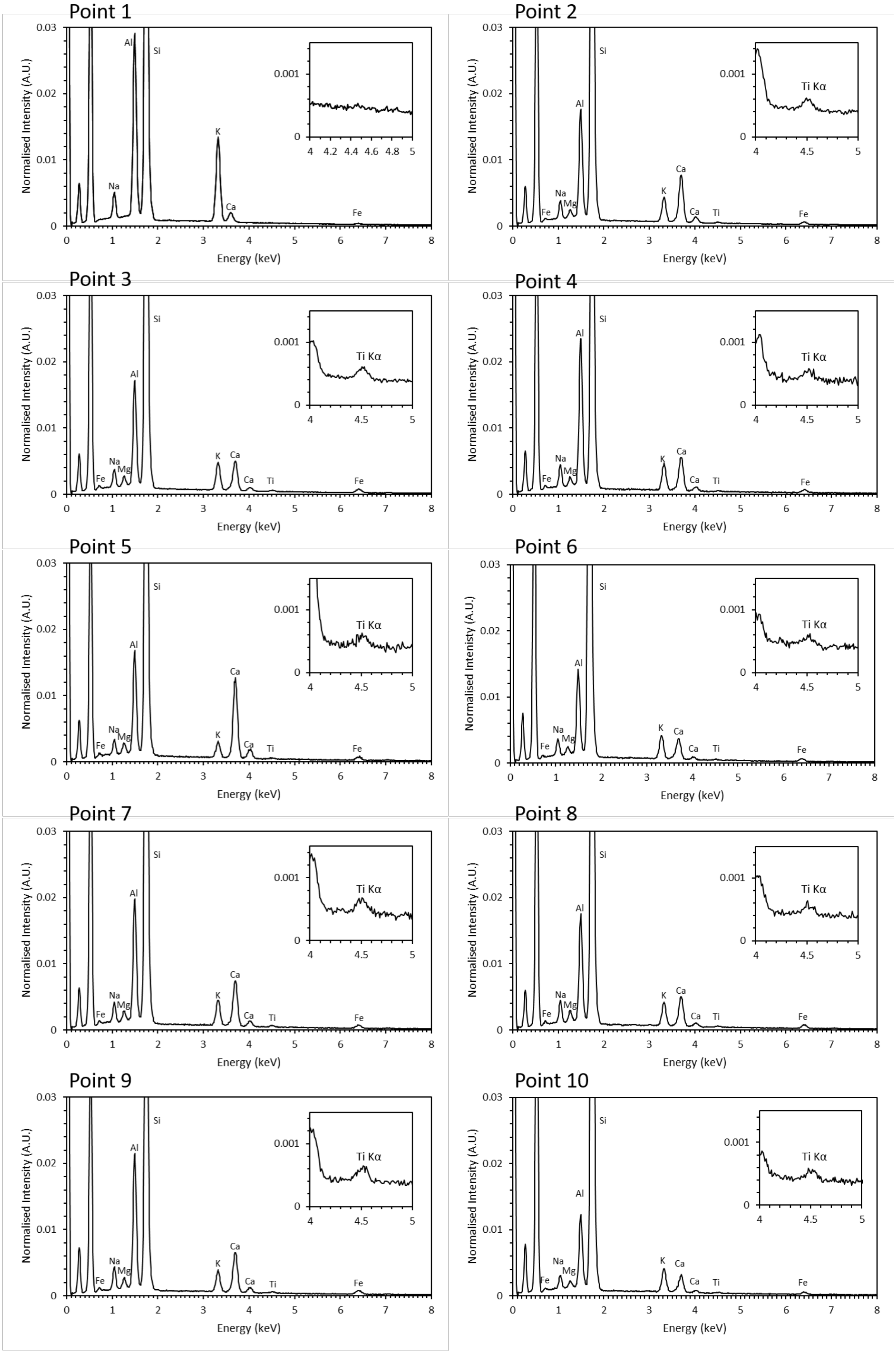

SF2: Normalised EDX spectra from point scans at locations indicated in SF1. Inset- Magnified Ti region.

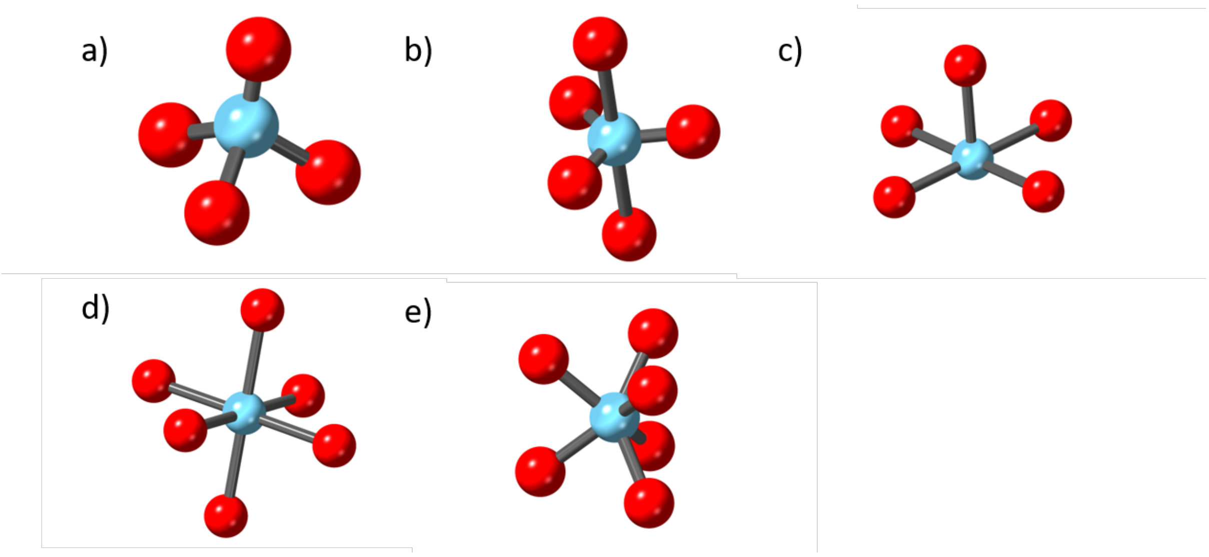

SF3: Possible four-, five- and six-fold coordination environments: a) Tetrahedral; b) Trigonal bi-pyramidal; c) Square pyramidal; d) Octahedral; e) Trigonal prismatic.

ST3: Pre-edge centroid height and energy of titanium standards, with co-ordination number and corresponding co-ordination environment. Adapted from Farges *et al* (1996).

Standard	Ti Co-ordination	Co-ordination environment	Pre-edge centroid position (eV)	Pre-edge centroid height
$Ni_{2.6}Ti_{0.7}O_4$	4	Tetrahedral	4969.7	0.94
$Ni_{2.4}Ti_{0.7}Si_{0.05}O_4$	4	Tetrahedral	4969.6	0.90
β- Ba_2TiO_4	4	Tetrahedral	4969.4	1.00
α- Ba_2TiO_4	4	Tetrahedral	4969.5	1.00
$CsAlTiO_4$	4	Tetrahedral	4969.7	0.90
Rb_2TiO_3	4	Tetrahedral	4969.6	1.00
$K_6Ti_2O_7$	4	Tetrahedral	4969.7	0.93
Y_2TiMoO_8	4	Tetrahedral	4969.9	0.74
Na_4TiO_4	4	Tetrahedral	4969.6	0.70
$Na_2TiOSiO_4$	5	Square pyramidal	4970.5	0.65
$KNaTiO_3$	5	Square pyramidal	4970.6	0.73
$Ba_2TiOSi_2O_7$	5	Square pyramidal	4970.6	0.67
$Sr_2TiOSi_2O_7$	5	Square pyramidal	4970.5	0.71
$Ba_2TiOGe_2O_7$	5	Square pyramidal	4970.6	0.64
$Na_2Ti_4O_9$	5/6	Square pyramidal/Octahedral	4970.5	0.43
$K_2Ti_4O_9$	5/6	Square pyramidal/Octahedral	4970.6	0.45
$Rb_2Ti_4O_9$	5/6	Square pyramidal/Octahedral	4970.5	0.47
$K_2Ti_2O_5$	5	Square pyramidal	4970.6	0.51
r-TiO_2	6	Octahedral	4971.6	0.22
a-TiO_2	6	Octahedral	4971.5	0.17
$CaTiO_3$	6	Octahedral	4971.6	0.11
$BaTiSi_3O_9$	6	Octahedral	4971.2	0.04
$KNa_2LiFe_2Ti_2Si_8O_{24}$	6	Octahedral	4971.1	0.32
$FeTiO_3$	6	Octahedral	4971.7	0.22
$CaTiSiO_5$	6	Octahedral	4971.4	0.18
$TiZrO_4$	6	Octahedral	4971.1	0.21
$Ca_3(Ti,Zr)_2(Si,Al_2)O_{12}$	6	Octahedral	4971.2	0.15
